# Effect of feeding rumen-protected methionine on productive and reproductive performance of dairy cows

**DOI:** 10.1371/journal.pone.0189117

**Published:** 2017-12-20

**Authors:** Mateus Z. Toledo, Giovanni M. Baez, Alvaro Garcia-Guerra, Nelson E. Lobos, Jerry N. Guenther, Eduardo Trevisol, Daniel Luchini, Randy D. Shaver, Milo C. Wiltbank

**Affiliations:** 1 Department of Dairy Science, University of Wisconsin-Madison, Madison, Wisconsin, Unites States of America; 2 Endocrinology and Reproductive Physiology Program, University of Wisconsin-Madison, Madison, WI, United States of America; 3 Adisseo USA Inc., Alpharetta, Georgia, Unites States of America; INIA, SPAIN

## Abstract

The objectives of this study were to evaluate the effects of daily top-dressing (individually feeding on the top of the total mixed ration) with rumen-protected methionine (**RPM**) from 30 ± 3 until 126 ± 3 Days in milk on productive and reproductive performance in lactating dairy cows. A total of 309 lactating dairy Holstein cows (138 primiparous and 171 multiparous) were randomly assigned to treatment diets containing either RPM (21.2 g of RPM + 38.8 g of dried distillers grain; 2.34% Methionine [**Met**] of metabolizable protein [**MP**]) or Control (**CON;** 60 g of dried distillers grain; 1.87% Met of MP). Plasma amino acids were evaluated at the time of artificial insemination (**AI**) and near pregnancy diagnosis. Milk production and milk composition were evaluated monthly. Pregnancy was diagnosed on Day 28 (by Pregnancy-specific protein B [**PSPB**]), 32, 47, and 61 (by ultrasound) and sizes of embryonic and amniotic vesicle were determined by ultrasound on Day 33 after AI. Feeding RPM increased plasma Met at 6, 9, 12, and 18 hours after top-dressing with a peak at 12 hours (52.4 vs 26.0 μM; P < 0.001) and returned to basal by 24 hours. Cows fed RPM had a small increase in milk protein percentage (3.08 vs 3.00%; P = 0.04) with no differences on milk yield and milk protein yield. Additionally, in multiparous cows, RPM feeding increased milk protein (3.03 vs 2.95%; P = 0.05) and fat (3.45 vs 3.14%; P = 0.01) percentages, although no effects were observed in primiparous cows. In multiparous cows fed RPM, pregnancy loss was lower between Days 28 to 61 (19.6 [10/51] vs. 6.1% [3/49]; P = 0.03) or between Days 32 to 61 (8.9 [4/45] vs. 0 [0/0] %; P = 0.03), although, there was no effect of treatment on pregnancy loss in primiparous cows. Consistent with data on pregnancy loss, RPM feeding increased embryonic abdominal diameter (P = 0.01) and volume (P = 0.009) and amniotic vesicle volume (P = 0.04) on Day 33 of pregnancy in multiparous cows but had no effect on embryonic size in primiparous cows. Thus, the increase in plasma Met concentrations after feeding RPM was sufficient to produce a small increase in milk protein percentage and to improve embryonic size and pregnancy maintenance in multiparous cows. Further studies are needed to confirm these responses and understand the biological mechanisms that underlie these responses as well as the timing and concentrations of circulating Met that are needed to produce this effect.

## Introduction

Nutritional deficiencies can reduce fertility [1–3], alter embryonic or fetal development at many stages of pregnancy [4–6], and even lead to pregnancy loss [7–9]. One nutritional element that may have an important role in reproduction in lactating dairy cattle is amino acid (**AA**) nutrition. Many AA are concentrated in the oviductal and uterine histotroph and in the amniotic and allantoic fluids, compared to circulating AA concentrations, and several investigators have postulated an important role for these elevated AA concentrations in normal embryonic and fetal development [10–12].

One essential AA that could potentially be limiting for reproduction in lactating dairy cows is methionine (**Met**). In mammals, a Met codon is used for initiation of most protein synthesis producing an essential role for this AA in all aspects of mammalian cellular functions [13–15]. Previous studies that evaluated effects of feeding rumen-protected methionine (**RPM**) on milk production demonstrated a consistent increase in milk protein percentage and generally milk protein yield [16–18]. For reproduction, previous studies have linked concentrations of Met with optimal early embryonic development [19–22]. A recent *in vivo* study demonstrated that feeding RPM in lactating dairy cattle produced dramatic alterations in gene expression in embryos, generally decreasing concentrations of mRNA in early embryos [21]. In addition, studies in both sheep [12, 23] and cattle [10, 11] have demonstrated that Met is concentrated in uterine and embryonic fluids, suggesting a role for elevated uterine Met in normal embryonic development and survival. In spite of these studies that link Met with milk production and reproductive processes, no previous studies have evaluated the effects of feeding RPM on fertility and pregnancy loss in lactating dairy cows.

The hypothesis for this study was that feeding RPM would enhance production and reproduction in lactating dairy cows. In order to maintain cow as the experimental unit, cows were supplemented with RPM or a vehicle control by daily top-dressing (individually feeding on the top of the total mixed ration). We specifically hypothesized that RPM feeding would increase plasma Met, and milk protein percentage and production, as observed in previous studies [16–18]. Further, we hypothesized that RPM feeding would accelerate embryonic development as measured by increased Pregnancy-specific protein B (**PSPB**) concentration and increased embryonic and amniotic vesicle sizes, and therefore there would be an increase in pregnancies per artificial insemination (P/AI) and reduced pregnancy loss. Our objectives were to evaluate the effects of daily top-dressing with RPM on circulating plasma AA, milk production and milk composition, embryo development, P/AI and pregnancy loss.

## Materials and methods

All procedures in this study including, hormonal treatments, blood collections, and ultrasonography were approved by the Animal Care and Use Committee of the College of Agriculture and Life Sciences, University of Wisconsin.

### Animals, management and enrollment in the experiment

Cows were housed in free stall facilities at a commercial dairy farm between November 2013 and May 2014. A total of 309 lactating dairy Holstein cows (138 primiparous and 171 multiparous) were fed once daily a diet (Table 1) at 8 a.m. for 5% refusal with *ad libitum* access to feed and water. The pens had feed-line head lockups (cows could be locked at the feed bunk at the time of feeding) and free stalls bedded with recycled manure. Throughout the experiment, cows were milked twice daily at approximately 12 hour intervals.

**Table 1:** Ingredient and chemical composition of the experimental diets.^1^.

Item	CON	RPM	± SD^2^
Ingredient, % of Dry Matter (**DM**)			
Corn silage	29.6	29.6	1.3
Alfalfa haylage	17.5	17.5	2.0
High-moisture shelled corn	18.2	18.2	0.9
Cottonseed whole	8.8	8.8	0.3
Barley malt sprouts	7.6	7.6	0.7
Canola meal	4.0	4.0	0.4
Soybean meal, heat processed^3^	3.6	3.6	0.0
Soybean meal	2.4	2.4	0.1
Starch corn	3.4	3.4	0.1
ProVAAL advantage^4^	0.6	0.6	0.0
Megamine L^5^	0.7	0.7	0.0
Urea	0.1	0.1	0.0
Beef tallow	0.5	0.5	0.0
Smartamine M^6^	---	0.10	0.00
Dried distillers grains	0.27	0.17	0.00
Mineral-vitamin mix	2.7	2.7	0.1
Chemical Composition			
DM, % of as fed	49.2	49.2	2.4
CP, % of DM	16.7	16.7	0.5
Ash, % of DM	6.3	6.3	0.2
aNDF, % of DM	31.5	31.5	1.5
Starch, % of DM	28.8	28.8	1.4
NFC, % of DM^7^	40.0	40.0	1.4
Fat, % of DM	6.2	6.2	0.2
MP supplied, g/d^8^	2516.8	2525.0	268.0
MP balance, g/d^8^	166.3	178.7	241.8
Hist, % of MP^8^	2.72	2.72	0.06
Hist, g/d^8^	68.8	68.9	6.5
Lys, % of MP^8^	6.95	6.92	0.10
Lys, g/d^8^	174.5	174.7	16.3
Met, % of MP^8^	1.87	2.34	0.03
Met, g/d^8^	46.9	59.0	5.4
Lys:Met ratio^8^	3.71	2.96	0.03

^1^Treatments: Control (CON) = feeding with 60 g of distillers grains (1.87% Met of MP); and Rumen-protected methionine (RPM) = 21.2 g of rumen-protected methionine (Smartamine M, 2.34% Met of MP) + 38.8 g of dried distillers grains.

^2^Standard deviation for ingredients and chemical composition during the experimental period.

^3^Soy Best (West Point, NE).

^4^Amino acid supplement [7.1% of Lys and 0.9% Met as % of DM (Perdue Agribusiness; Salisbury, MD: https://www.perdueagribusiness.com/precision-dairy-nutrition)].

^5^Rumen-protected Lys (Arm & Hammer Animal Nutrition; Church & Dwight Co., Inc., Ewing, NJ)

^6^Rumen-protected methionine (Adisseo Inc., Alpharetta, GA).

^7^NFC (CNCPS calculation): 100 - ([% NDF—% NDF-CP) + % CP + % fat + % ash)

^8^Estimated supply of MP and g of Met, Lys and His using Cornell Net Carbohydrate and Protein System v. 6.1 as implemented by AMTS.Cattle.Professional v. 3.4.7 (2013, AMTS LLC, Groton, NY).

**Fig 1:**
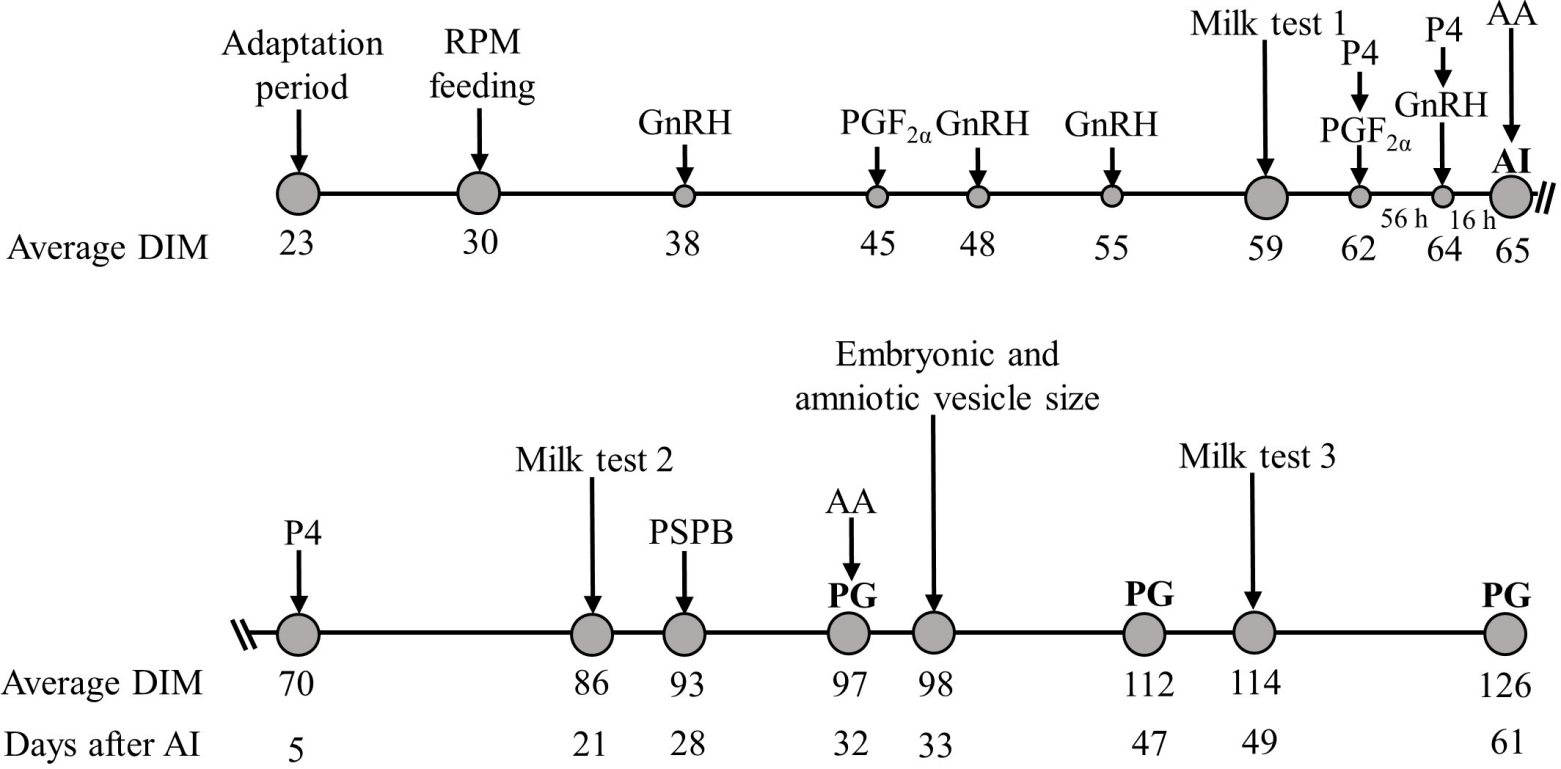
Schematic representation of experimental protocol showing the period of adaptation (7 days), 96 days of feeding (CON vs. RPM), the reproductive protocol used, and each measurement taken throughout the experiment. The Double Ovsynch protocol was used to synchronize ovulation in all cows and timed AI was performed on Day 65 ± 3. Pregnancy diagnosis (PG) were performed on Days 32, 47, and 61 after AI. RPM feeding started on Day 30 ± 3 and continued until a cow was diagnosed not pregnant on Day 32 or until Day 61 after AI. Blood samples were collected on Day of last PGF_2α_ and GnRH and on Day 5 and 28 after AI to analyze progesterone (P4) concentrations, on the week of AI and first PG to analyze AA concentrations, and on Day 28 after AI to analyze Pregnancy specific-protein B (PSPB) concentrations. Ultrasound videos were made on Day 33 after AI to evaluate embryonic and amniotic vesicle size. Milk tests were performed at (58 ± 9; mean ± SD), 73 to 100 (87 ± 9), and 101 to 128 (115 ± 9) DIM.

The complete sampling schedule during the experimental period is shown in Fig 1. Cows were moved to the experimental pen at 23 ± 3 days in milk (**DIM**), had one week of adaptation prior to experimental treatments, and were evaluated for proficiency at locking up and consuming the treatment (CON treatment provided for all cows during the adaptation period). Cows were locked up at the time of feeding, and had approximately 45 minutes to consume the supplement. On the Day of enrollment, cows had to meet the following requirements: no previous artificial insemination (**AI**), no abortion, no lameness, and a body condition score (BCS) ≥ 2.25. Cows were scored for body condition on a scale of 1 (thin) to 5 (obese), using increments of 0.25; [24]. During the experiment, 382 cows were moved into the pen study, and evaluated during the adaptation period. A total of 13 cows were not enrolled in the study for the following reasons: not adapting to top-dressing protocol (n = 4), not locking up (n = 2), and illness (mastitis, pneumonia, lameness, or uterine infection; n = 7). In total, 369 cows were evaluated during the adaptation period and enrolled in the study, but 60 cows were removed due to lack of consumption of supplement for 5 days (RPM = 6), movement to the sick pen for any reason (mastitis, pneumonia, lameness and uterine infection) without return to the experimental pen within 5 days (CON = 20; RPM = 27), sold (CON = 3; RPM = 1), missed one hormonal treatment (RPM = 1), or not inseminated (RPM = 2).

### Treatments and AI

Weekly, a cohort of cows at 30 ± 3 DIM were enrolled in the study blocked by parity (multiparous or primiparous) and assigned randomly to one of two treatments: (1) Rumen-protected methionine (**RPM**), diet formulated to deliver 2525.0 g of metabolizable protein (**MP**) with 6.92% Lysine (**Lys**), 2.72% Histidine (**His**) and 2.34% Met as a % of MP and a Lys:Met ratio of 2.96; (2) Control (**CON**), same basal diet, but formulated to deliver 2516.8 g of MP and contained 6.95% Lys, 2.72% His and 1.87% Met %MP and a Lys:Met ratio of 3.71. The diets were formulated using Cornell Net Carbohydrate and Protein System v. 6.1 [25] as implemented by AMTS.Cattle.Professional v. 3.4.7 (2013, AMTS LLC, Groton, NY). Our control diet is in agreement with Dairy NRC (2001), for commonly-fed diets that have not been supplemented with RPM and the target dietary estimated levels of Met in the RPM treatment and Lys and His for both treatments are based on NRC recommendations [26]. Both diets contained 16.7% ± 0.5 crude protein (CP; DM basis) and similar concentrations of energy, fiber, starch, fat, macro- and micro-minerals, and vitamins (Table 1; NRC, 2001). Dry matter intake was not recorded for individual cows. Cows in the CON group received once daily top-dressing of placebo containing 60 g of dried distillers grain. The RPM cows received 60 g of a mixture containing 38.8 g of dried distillers grain and 21.2 g of RPM (Smartamine M; Adisseo Inc). All cows were synchronized using the Double Ovsynch protocol, as previously described [27–29] using a PGF_2α_ analog (Cloprostenol sodium; 250 mcg/ml; Estroplan) and GnRH (Gonadorelin acetate; 100 mcg/ml; Gonabreed) provided by Parnell (Overland Park, KS). Briefly, cows were treated with a presynchronization Ovsynch (GnRH at 38 ± 3 DIM, 7 days later with PGF_2α_, and 3 days later with GnRH) and 7 days later they were synchronized with Ovsynch-56 and received fixed time AI (GnRH, 7 days later PGF_2α_, 56 hours later GnRH, followed 16 hours later by fixed time AI at 65 ± 3 DIM). The period of feeding continued until 61 days pregnant (126 ± 3 DIM). The study ended for a cow if she reached 61 days of pregnancy or was found to be not pregnant at the first ultrasound pregnancy diagnosis (32 days after AI or 97 ± 3 DIM). Thus, a cow remained in the study for 67 days if she was not pregnant on Day 32 after AI or for 96 days if she was diagnosed pregnant on day 32 (until 61 days of pregnancy).

### Feed and milk samples

Samples of TMR were collected weekly and then composited by month. All samples for determination of nutrient composition were dried at 60°C for 48 hours in a forced-air oven to determine dry matter (DM) content, ground to pass through a 1-mm Wiley mill screen (Arthur H. Thomas, Swedesboro, NJ), and stored in sealed plastic containers at room temperature until composited monthly and sent to Dairyland Laboratories Inc. (Arcadia, WI) for nutrient analysis. All samples were analyzed for DM, organic matter (OM; method 942.05; AOAC International, 2012), CP (method 990.03; AOAC International, 2012), ether extract (method 2003.05; AOAC International, 2012), neutral detergent fiber (NDF) using α-amylase and sodium sulfite [30], and starch [31] using a YSI Biochemistry Analyzer (YSI Inc., Yellow Springs, OH). Milk volume was recorded and sampled monthly for analysis of fat and protein percentages and somatic cell count (AgSource Milk Analysis Laboratory, Menomonie, WI) using a Foss FT6000 (Foss Electric, Hillerod, Denmark). Milk test results for each cow were assigned to a specific milk test based on DIM prior to statistical analysis. The first, second, and third milk tests ranged from 45 to 72 (58 ± 9; mean ± SD), 73 to 100 (87 ± 9), and 101 to 128 (115 ± 9) DIM, respectively. If cows were on treatment for less than 15 days, milk tests were not used in the analysis. Based on milk sample analysis, the ECM and 3.5% FCM were calculated according to NRC (2001) equations: ECM = [0.3246 x milk yield (kg)] + [12.86 x fat yield (kg)] + [7.04 x protein yield (kg)] and 3.5% FCM = (0.432 x milk yield (kg) + (16.23 * fat yield (kg) [26].

### Blood sampling

Blood samples were collected to determine concentrations of serum progesterone (**P4**), PSPB, and plasma AA. For all cows, blood samples were collected during the synchronization protocol on Day of final PGF_2α_, last GnRH, and at 5 and 28 days after AI at the time of feeding for evaluation of circulating P4 and at Day 28 after AI for PSPB concentration. For the first AA analyses to determine the timing of the increase in circulating Met after RPM feeding, a subset of 20 cows (8 primiparous and 12 multiparous) on the week of pregnancy diagnosis (97 ± 3 DIM; 67 days after start of treatments) had blood samples collected at 0, 3, 6, 9, 12, 18 and 24 hours after feeding and top-dressing with RPM. Samples from 4 cows were pooled for analyses, yielding a total of 5 composite samples that were analyzed, 2 CON and 3 RPM. In a second analysis, another subset of 40 cows (16 primiparous and 24 multiparous), also near pregnancy diagnosis, had blood samples collected at 12 hours after feeding and RPM top-dressing. Samples from 4 cows were again pooled for analysis of AA to yield 4 composite samples from CON (2-primiparous and 2-multiparous) and 6 composite samples from RPM cows (3-primiparous and 3-multiparous). In addition, a third analysis was performed with data from the first and second analysis combined with another subset of cows (n = 65, 35 primiparous and 30 multiparous) near AI with blood collection at 24 hours after feeding and RPM top-dressing. Samples from 4 to 5 cows were again pooled to yield 7 composite samples from CON (4-primiparous and 3-multiparous) and 7 composite samples from RPM (4-primiparous and 3-multiparous). For this last analysis, CON (0, 12, 24 hours from all subsets) had a total of 17 composite samples (9-primiparous and 8 multiparous) and RPM had a total of 13 (6-primiparous and 7 multiparous) and 9 (4-primiparous and 5-multiparous) composite samples at 24 hour (0 and 24 hour pooled) and 12 hour, respectively. This last analysis allowed a test of the effect of parity under CON conditions, with no interference of feeding RPM.

Blood collection was performed via puncture of the coccygeal blood vessels into evacuated tubes (Vacuette, 9 ml; Greiner Bio-One North America Inc., Monroe, NC) for determination of circulating P4 and PSPB or into evacuated tubes containing EDTA (Vacutainer 10 ml; Becton Dickinson, Franklin Lakes, NJ) for AA concentrations. After collection, tubes were placed immediately on ice and transported to the laboratory. Blood samples were centrifuged at 1900 x g at 4°C for 20 min and serum or plasma was isolated into vials, frozen and stored at -20°C until assayed for P4, PSPB, or plasma AA concentrations.

### Plasma AA analyses

The concentrations of plasma AA (except arginine [Arg], cysteine [Cys], tryptophan [Trp], glutamine [Gln], asparagine [Asn]; Arg and Cys could not be obtained with GC-FID and Trp, Gln, Asn require a separated standard kit) were determined in plasma by analyzing the free AA concentration using gas chromatography (Shimadzu gas chromatographer model GC-2010 plus) and a commercial kit (EZ:faast™ GC-FID Physiological, Phenomenex) [32, 33]. Briefly, AA were removed by solid phase extraction, then derivatized by using chlorophormate [34], separated by a mid-polar capillary column, and detected by flame ionization. Quantification is based on area under the curve by using the internal standard method (norvaline). Seven standards (3.125, 6.25, 12.5, 25, 50, 100, and 200 ng/ml) and a blank were analyzed and a standard curve was constructed based on these standards. Samples were analyzed in singlet but with two GC injections for each sample.

### PSPB and P4 assay

To analyze the concentrations of PSPB, a commercially available quantitative ELISA assay (Biopryn, BioTracking LLC, and Moscow, ID) was used with a few modifications. The BioPRYN assay kit contains all the standards and controls for analysis of each 96-well plate. In addition to the standards (0.125, 0.25, 0.5, 1.0, 2.0, and 4.0 ng/ml), a blank (assay buffer) were used for development of the standard curve. All samples were first assayed in singlet. Samples in which the PSPB assay results did not match the expected results based on pregnancy status as determined by ultrasound (Pregnant and PSPB < 2 ng/ml (n = 13) or not pregnant and PSPB > 0.5 ng/ml (n = 36) were re-run in duplicate. All values for cows carrying twins (CON = 3; RPM = 1) were analyzed separately from cows carrying single calves. Two quality control samples were analyzed in duplicate on each plate in order to calculate inter- and intra-assay coefficient of variation (**CV)**. The inter-assay CV was 5.1% and the intra-assay CV was 2.1% for the 7 plates. The assay for P4 was done with serum using a solid phase radioimmunoassay kit (Diagnostic Products Corporation, Los Angeles, CA) with no extraction. Average intra and inter-assay CV for the P4 assays were 4.0% and 6.4%.

### Ovarian ultrasonography, ovulatory responses, and pregnancy diagnosis

Ultrasonography evaluations of the ovaries were performed using a portable ultrasound fitted with a 7.5 MHz linear-array transducer (Ibex Pro; E. I. Medical Imaging, Loveland, CO) during the protocol on the Day of final PGF_2α_, last GnRH, and 5 days after AI to determine the number of corpora lutea, the largest follicle, ovulation, and synchronization to the protocol. All cows that had P4 concentrations below 1.0 ng/ml on Day of the final PGF_2α_, greater than 0.7 ng/ml on Day of the last GnRH, or below 0.5 ng/ml at 5 days after AI were considered to be not synchronized by the protocol [35] and were not used in analysis of effect of treatment on reproductive values. Overall, a total of 7.8% (n = 24/309) of cows were removed from the study due to lack of synchronization. Pregnancy diagnosis by ultrasonography was performed by detecting a viable conceptus on Day 32 after AI. All cows diagnosed as pregnant on Day 32 were reexamined on Day 47, and 61. Pregnancy per AI was defined as the number of cows pregnant on Days 32, 47, or 61 after AI divided by the total number of cows that received AI. Pregnancy loss was determined for cows that were pregnant on Day 28 or 32, but not pregnant at later pregnancy diagnoses, such as on Day 61. All cows carrying twins ([CON = 3; RPM = 1) were included in the fertility responses (all were diagnosed pregnant with twins on Days 32, 47, and 61).

### Embryo and amniotic vesicle size measurements

Cows diagnosed pregnant on Day 32 were reexamined by transrectal ultrasonography using a portable ultrasound 7.5 MHz linear-array transducer (Ibex Pro; E. I. Medical Imaging, Loveland, CO) on Day 33 to determine amniotic vesicle and embryo size similar to previous studies [36, 37] with some modifications. Each video was recorded for 16 seconds. At a later time, the videos were analyzed frame by frame by two independent people to determine the optimal position and orientation of the conceptus to measure embryo crown-rump length and abdominal diameter. In addition, the widest longitudinal and transverse diameters of the amniotic vesicle were determined. The ellipsoid volume of the amniotic vesicle or embryo was calculated using the formula: V = 4/3*π (LD/2*TD/2*TD/2) such that: V = volume, LD = longitudinal diameter or crown-rump length and TD = transversal diameter or abdominal diameter. All videos were analyzed by two individuals using the open-source image processing software, ImageJ (National Institute of Health, Bethesda, MD; http://rsb.info.nih.gov/ij/index.html) and the mean was used for analysis. The CV for the two independent measurements was calculated for each evaluator and videos with CV above 20% were reanalyzed. All videos with CV greater than 20% or unclear videos with inaccurate visualization of amniotic vesicle (CON, n = 16; RPM = 8) or embryo (CON = 10; RPM = 6) were eliminated from analysis. All cows carrying twins (CON = 3; RPM = 1) were not included in the analysis.

### Statistical analyses

The experimental design was a randomized complete block design with parity (primiparous vs. multiparous) as the blocking factor. All statistical analyses were performed using SAS software, version 9.4 (SAS Institute Inc., Cary, NC). A significant difference between treatment groups was considered when P ≤ 0.05, whereas differences between P > 0.05 and P ≤ 0.10 were considered a tendency. Data are presented as means ± standard error of the mean, obtained using PROC MEANS of SAS.

Binary outcomes such as P/AI, pregnancy loss, and proportion of cows synchronized at the time of final PGF_2α_, at last GnRH, and 5 Day after AI were analyzed by logistic regression using PROC LOGISTIC of SAS. Treatment and parity were included in the model as a fixed effect. For parameters in which the frequency of one of the outcomes was extremely low (e.g. pregnancy loss) exact logistic regression was performed. A one-tailed comparison was used for fertility outcomes (P/AI and pregnancy loss) as we hypothesized that feeding RPM would improve fertility outcomes.

Plasma Met, Lys, and His concentrations were compared between treatments and over time by ANOVA for repeated measures using PROC MIXED of SAS. Treatment, time, and their interaction were included in the model and an autoregressive covariance structure used to account for repeated measurements from the same animal. Preplanned comparisons of treatment means at each time point were performed using the LSD method. Plasma AA concentrations at 12 hour and 24 hour after top-dressing were analyzed between groups by ANOVA using PROC MIXED of SAS for comparisons between treatment and parity.

Production variables were analyzed to evaluate effects of treatment and parity over time by ANOVA for repeated measures using PROC MIXED of SAS. An autoregressive covariance structure was used to account for repeated measurements from the same animal. Treatment, parity, milk test based on DIM, and their interactions were included in the model. Only cows that had production data for all 3 milk tests were used for this analysis. The assumptions of normality and homogeneity of variance were evaluated in all models using residuals plots and outcomes were transformed, if necessary.

Serum P4 concentrations at the time of final PGF_2α_, at last GnRH, and 5 days after AI were compared between treatments by ANOVA using PROC MIXED of SAS. The concentration of P4 and PSPB on Day 28 after AI were compared between groups by ANOVA using PROC MIXED of SAS. Treatment, pregnancy status, and parity were included in the model as fixed effects and interactions were evaluated and retained if significant. The variable pregnancy status described three potential outcomes: animals that were not pregnant, animals that became pregnant on Day 28 and maintained the pregnancy until Day 61, and animals that became pregnant on Day 28 but lost the pregnancy between Day 28 and 61. Model assumptions were analyzed with residual plots and outcomes showing deviations from the assumption of normality and/or homogeneity of variance were log transformed.

Amniotic vesicle and embryo measurements were compared between groups by ANOVA using PROC MIXED of SAS. Treatment and parity were included in the model as fixed effects and comparisons between treatments for each parity were carried out using LSD.

A significant difference between treatment groups was considered when P ≤ 0.05, whereas differences between P > 0.05 and P ≤ 0.10 were considered a tendency.

## Results

### Plasma AA concentrations

**Fig 2:**
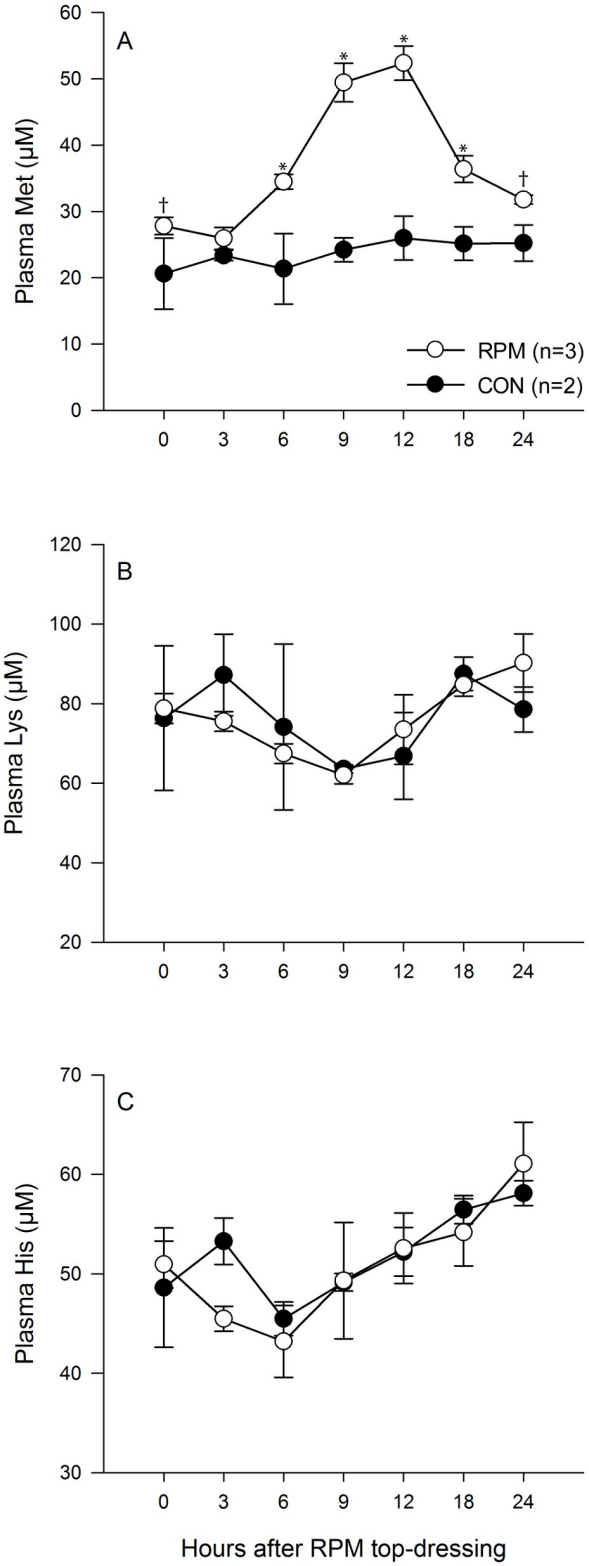
**Concentrations of plasma Met (A), Lys (B), and His (C) concentrations after rumen-protected methionine (RPM) top-dressing for CON (●; n = 2 composite samples from 8 cows) or RPM (○ n = 3 composite samples from 12 cows).** For Panel A, there was an effect of treatment (P < 0.001), time (P < 0.001), and an interaction of treatment x time (P = 0.002). For Panel B, effect of treatment (P = 0.99), time (P = 0.08) and the interaction of treatment x time (P = 0.71). For Panel C, effect of treatment (P = 0.85), time (P = 0.001) and the interaction of treatment x time (P = 0.03). An asterisk (*) indicates that mean values differ (P < 0.05) within a specific time. A tendency is indicated by a dagger (†) (0.05 < P ≤ 0.10).

The first analysis of AA was done to obtain a temporal profile for the changes in plasma Met, Lys, and His after feeding and top-dressing the RPM (Fig 2). After top-dressing with RPM, the plasma concentrations of Met were at basal concentrations at 0 and 3 hours. However, the plasma Met was greater in RPM than in CON at 6 hour (34.5 ± 1.1 vs 21.4 ± 5.3 μM; P = 0.001) and 9 hour (49.5 ± 2.9 vs 24.2 ± 1.8 μM; P < 0.001) after top-dressing, and reached a peak concentration at 12 hour (52.4 ± 2.6 vs 26.0 ± 3.3 μM; P < 0.001). After 18 hour, plasma Met concentrations decreased in RPM cows, but remained higher in RPM than in CON (36.4 ± 2.0 vs 25.2 ± 2.5 μM; P = 0.008). Subsequently, plasma Met returned to basal concentrations in RPM at 24 hour. Plasma Lys and His were similar in RPM or CON cows at all times after feeding.

In a second analysis of AA, cows were sampled only at 12 hour after feeding and top-dressing with RPM. Cows fed with RPM had greater plasma Met (42.5 ± 2.8 vs 23.7 ± 0.3μM; P = 0.001), but Lys and His were not different between treatments. In addition, cows fed RPM had increased Met in both primiparous (46.4 ± 3.3 vs 23.9 ± 0.7 μM; P = 0.01) and multiparous (38.7 ± 3.5 vs 23.5 ± 0.1 μM; P = 0.05) cows, but Lys and His remained similar between treatments in the two parity groups. No changes were observed in other AA concentrations at 12 hour after RPM feeding (Table 2).

**Table 2:** Plasma amino acids (AA) concentrations at 12 hour after rumen-protected methionine (RPM) feeding.

	Plasma AA concentration, μM^1^^,^^2^^,^^3^		
AA	CON (n = 4)^4^	RPM (n = 6)^4^	P-value^5^
Alanine	305.7 ± 22.8	315.6 ± 13.5	0.70
Aspartic acid	6.6 ± 0.7	6.1 ± 0.5	0.51
Glutamic acid	63.7 ± 4.9	68.4 ± 4.9	0.53
Glycine	286.0 ± 16.4	302.9 ± 18.0	0.53
Histidine	47.6 ± 4.1	42.4 ± 1.1	0.29
Isoleucine	123.3 ±3.7	121.1 ± 5.6	0.78
Leucine	123.0 ± 9.2	123.6 ± 5.2	0.95
Lysine	69.0 ± 9.2	68.1 ± 2.7	0.93
Methionine	23.7 ± 0.3	42.5 ± 2.8	0.001
Phenylalanine	38.7 ± 1.7	40.2 ± 1.1	0.46
Proline	99.3 ± 3.9	103.3 ± 4.7	0.56
Serine	130.5 ± 9.9	130.8 ± 3.4	0.98
Threonine	127.9 ± 9.9	143.0 ± 5.8	0.19
Tyrosine	43.6 ± 2.9	47.1 ± 1.5	0.26
Valine	260.8 ± 13.0	260.0 ± 10.2	0.96

^1^Treatments: Control (CON) = feeding with 60 g of dried distillers grain (1.87% Met of MP); and Rumen-protected methionine (RPM) = 21.2 g of rumen-protected methionine (Smartamine M, 2.34% Met of MP) + 38.8 g of dried distillers grain.

^2^All AA considered essential and nonessential. Concentrations of arginine, cysteine tryptophan, glutamine, and asparagine, were not obtained.

^3^Data are presented as mean ± SEM.

^4^CON = 4 composites (16 cows, 8 primiparous and 8 multiparous); RPM = 6 composites (24 cows, 12 primiparous and 12 multiparous).

^5^P-values for comparisons between CON and RPM

**Fig 3:**
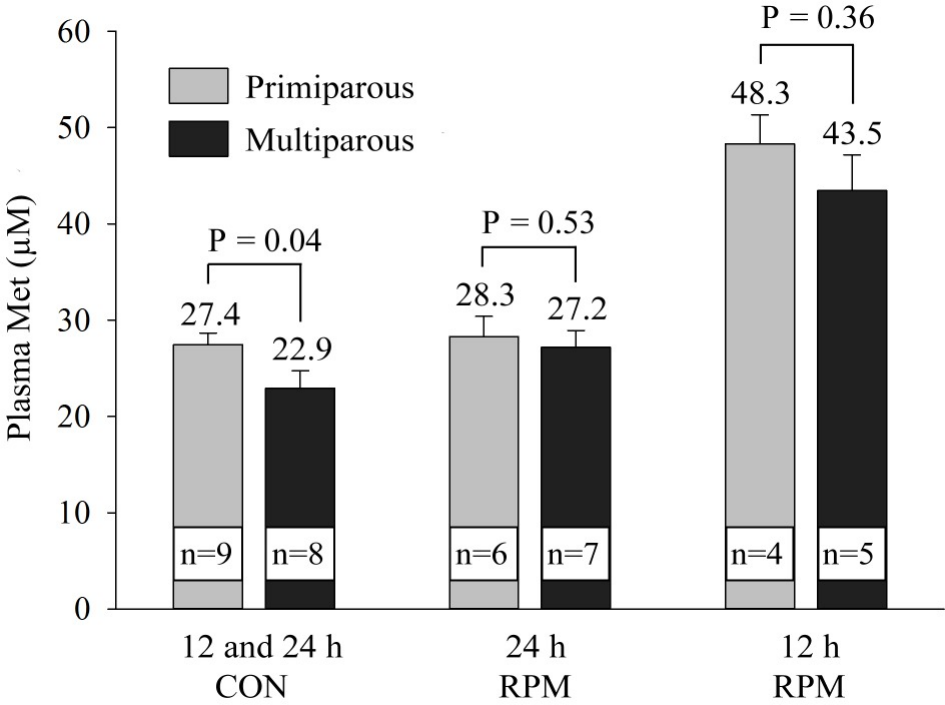
Concentrations of plasma Met analyzed by parity for CON cows at 12 and 24 hours (average of all values), RPM at 12 hour, and RPM at 24 hour after feeding and RPM feeding. CON at 12 and 24 hours (primiparous = 9 composites; multiparous = 8 composites); RPM at 24 hour (primiparous = 6 composites; multiparous = 7 composites); RPM at 12 hour (primiparous = 4 composites; multiparous = 5 composites). Each composite contains 4 to 5 cows.

Results from these two analyses were combined to evaluate overall parity effects at 12 and 24 hours after RPM feeding (Fig 3). In cows fed RPM, there was no effect of parity on plasma Met, Lys, or His at 12 and 24 hours after RPM feeding (Fig 3). In CON cows, the two times were combined (12 and 24 hours) and there was an overall effect of parity on plasma Met concentrations with, multiparous cows having lower (P = 0.04) plasma Met concentration compared to primiparous cows (22.9 ± 1.2 vs 27.4 ± 1.8 μM, respectively).

### Milk production and composition

Table 3 shows the effect of RPM feeding on milk production and composition by parity. Only cows that were evaluated in all 3 milk tests were utilized in the analysis.

**Table 3:** Effect of rumen-protected methionine feeding (RPM) on production variables analyzed for all cows and by parity.

	Treatment^1^				Parity/Treatment^1^							
	Overall				Primiparous				Multiparous			
Item^3^	CON	RPM	SEM^4^	P^2^	CON	RPM	SEM4	P	CON	RPM	SEM4	P
n^5^	60	62			26	30			34	32		
Milk Yield, kg/d	41.6	39.9	0.8	0.32	34.5	34.2	0.8	0.90	47.3	45.3	0.9	0.18
ECM, kg/d	39.9	39.5	0.7	0.92	34.8	34.3	0.8	0.96	44.0	44.4	0.9	0.85
3.5% FCM, kg/d	40.2	39.9	0.7	0.90	34.4	34.6	0.7	0.91	44.3	44.9	0.9	0.76
Fat, %	3.33	3.52	0.05	0.07	3.58	3.60	0.07	0.91	3.14	3.45	0.07	0.01
Fat, kg/d	1.37	1.40	0.03	0.42	1.23	1.22	0.03	0.94	1.47	1.56	0.04	0.20
Protein, %	3.00	3.08	0.02	0.04	3.07	3.12	0.03	0.32	2.95	3.03	0.02	0.05
Protein, kg/d	1.24	1.22	0.02	0.87	1.06	1.06	0.02	0.83	1.39	1.37	0.03	0.64
SCC x 10^3^, cells/ml	124.6	78.9	27.6	0.37	165.0	86.8	57.8	0.66	93.5	71.6	24.4	0.39

^1^Treatments: Control (CON) = feeding with 60 g of dried distillers grain (1.87% MET of MP); and Rumen-protected methionine (RPM) = 21.2 g of rumen-protected methionine (Smartamine M, 2.34% MET of MP) + 38.8 g of dried distillers grain.

^2^P = effect of treatment. All variables are affected by parity. The interaction between treatment and parity was not significant. Milk fat percentage, milk protein percentage, milk fat yield, milk protein yield and 3.5% FCM were affected by milk test based on DIM (P < 0.05). The interaction of treatment and milk test was not significant.

^3^Data are presented as mean of the 3 milk tests.

^4^Greatest SEM.

^5^Each cow had 3 milk tests. Milk test 3 is represented only by pregnant cows. Thus, all cows in this analysis are also pregnant.

Feeding RPM tended to increase milk fat percentage (3.52 vs 3.33%, SEM = 0.05; P = 0.07) and increased milk protein percentage (3.08 vs 3.00%, SEM = 0.02; P = 0.04) compared to CON, although there was no effect on milk protein yield (1.22 vs 1.24 kg/d, SEM = 0.02) and fat yield (1.40 vs 1.37 kg/d, SEM = 0.03) and these effects were independent of parity. Moreover, compared to primiparous cows, multiparous cows had greater (P < 0.001) milk yield (46.6 ± vs 34.6 ± kg/d, SEM = 0.8), ECM (44.9 vs 34.5 kg/d, SEM = 0.9), and 3.5% FCM (45.4 vs 34.9 kg/d, SEM = 0.9). In addition, multiparous cows had lower (P < 0.001) milk fat percentage (3.36 vs 3.57%, SEM = 0.07) and milk protein percentage (3.00 vs 3.09%, SEM = 0.02), but greater milk fat yield (1.56 vs 1.23 kg/d, SEM = 0.04), milk protein yield (1.39 vs 1.06 kg/d, SEM = 0.02), and SCC (124.4 vs 68.4 x 10^3^ cells/ml, SEM = 32.6). When production data were analyzed by parity, no effects of treatment were observed on any of the variables in primiparous. In contrast, in multiparous cows, no effects were observed on milk yield, ECM, 3.5% FCM, milk protein yield, milk fat yield, SCC, but cows fed RPM had greater milk fat percentage (3.14 vs 3.45%, SEM = 0.07; P = 0.01) and milk protein percentage 3.03 vs 2.95%, SEM = 0.02; P = 0.05).

### Determination of synchronization using circulating P4

Overall, there were 7.8% of cows that were considered non-synchronized and these cows had reduced (P < 0.001) P/AI compared to cows that were correctly synchronized (60.0% vs 12.5%; Table 4). There was no effect of treatment (CON = 91.5% [140/153]; RPM = 92.9% [145/156]) or lactation (primiparous = 91.3% [126/138]; multiparous = 93.0% [159/171]) on synchronization. A total of 9 cows (4 CON [2 primiparous and 2 multiparous]; 5 RPM [3 primiparous and 2 multiparous]) had low P4 at the time of PGF_2α_ (< 1.0 ng/ml), and these cows had reduced (P = 0.01) P/AI compared to cows with greater circulating P4. A total of 9 cows (5 CON [1 primiparous and 4 multiparous]; 4 RPM [2 primiparous and 2 multiparous]) did not have decreased circulating P4 at time of last GnRH (> 0.7 ng/ml) and these cows had reduced (P = 0.008) fertility compared with cows with lower circulating P4. On Day 5 after AI, a total of 6 cows (4 CON [2 primiparous and 2 multiparous]; 2 RPM [2 primiparous]) did not have an increase in circulating P4 (< 0.5 ng/ml) after AI and these cows had reduced P/AI (P = 0.05) compared to cows with greater circulating P4. There was no effect of treatment and lactation on P/AI for synchronized and non-synchronized cows. Circulating P4 on Day of PGF_2α_ (7.1 vs 7.3 ng/ml, SEM = 0.2), at the time of last GnRH (0.3 vs 0.3 ng/ml, SEM = 0.01) and on Day 5 after AI (1.6 vs 1.7 ng/ml, SEM = 0.1) were similar for CON vs RPM.

**Table 4:** Determination of synchronization based on progesterone (P4) concentrations. Cows were considered to not be synchronized if they had P4 < 1.0 ng/mL on Day of PGF_2α_, P4 > 0.7 ng/mL on Day of last GnRH, or P4 < 0.5 ng/mL on Day 5 after AI.

	Cows		Pregnancies/AI (P/AI)		
Day of	Not Synch^1^	Synch^2^	Not Synch^1^	Synch^2^	P^3^
PGF_2α_ (< 1.0 ng/ml)	2.9%	97.1%	11.1%	57.5%	0.01
	(9/308)	(299/308)	(1/9)	(172/299)	
Last GnRH (> 0.7 ng/ml)	3.2%	96.8%	10.0%	57.9%	0.008
	(10/309)	(299/309)	(1/10)	(173/299)	
Day 5 after AI (< 0.5 ng/ml)	2.0%	96.8%	25.0%	57.8%	0.05
	(8/304)	(296/304)	(2/8)	(171/296)	
Overall^4^	7.8%	92.2%	12.5%	60.0%	< 0.001
	(24/309)	(285/309)	(3/24)	(171/285)	

^1^Not Synch = cows not synchronized.

^2^Synch = cows synchronized.

^3^P–value for comparisons of P/AI between not synchronized cows and synchronized cows.

^4^If a cow was not synchronized by any of the 3 criteria then it was not included in the overall value.

### Analyses of circulating PSPB and P4 on Day 28 after AI

Overall, serum PSPB concentrations on Day 28 after AI (Table 5) were greater (P < 0.001) for cows diagnosed pregnant at all evaluations (Days 28, 32 and 61) compared to cows diagnosed non-pregnant on Day 28 (3.15 ± 0.07 vs 0.32 ± 0.02 ng/ml). In addition, the PSPB concentrations in cows detected pregnant on Day 28 was predictive of pregnancy maintenance or loss since cows that lost their pregnancy by Day 61 had lower PSPB concentrations (2.65 ± 0.20 ng/mL) than cows that maintained pregnancy to Day 61 (3.15 ± 0.07). The cut-off to determine pregnant cows on Day 28 after AI was established by the lowest PSPB concentration in a cow that was found to be pregnant on Day 32 using the ultrasound examination (1.37 ng/ml). A total of 16 cows were pregnant on Day 28, but not on d 32. To assure that the PSPB values were not spuriously high from a previous pregnancy, all of these cows were evaluated for PSPB concentration on Day 5 after AI and all cows were found to be below the pregnancy value on Day 5 and the Day 5 value was lower (P < 0.001) than the PSPB concentration on Day 28 after AI (0.55 ± 0.03 vs 2.33 ± 0.22 ng/ml). In addition, analyzing only pregnant cows on Day 28, primiparous cows were found to have greater (P = 0.005) PSPB concentration compared to multiparous cows (3.30 ± 0.11 vs 2.90 ± 0.08).

**Table 5:** Effect of rumen-protected methionine (RPM) feeding on Pregnancy-specific protein (PSPB) and progesterone (P4) concentrations on Day 28 in cows not pregnant, pregnant on Days 28, 32, and 61, or pregnant on Day 28 that subsequently lost their pregnancy.

		Pregnancy status	
Item/Treatment^1^^,^^2^	Not Pregnant	Pregnant on Day 28, 32 and 61	Pregnant on Day 28 but not on Day 61
PSPB (Day 28, ng/ml)			
CON	0.32 ± 0.04 (48)	3.27 ± 0.12 (72)	2.71 ± 0.28 (15)
RPM	0.32 ± 0.03 (48)	3.05 ± 0.09 (80)	2.54 ± 0.33 (9)
P-value^3^	0.90	0.22	0.55
Overall^4^	0.32 ± 0.02 (96)^a^	3.15 ± 0.07 (152)^b^	2.65 ± 0.20 (24)^c^
P4 (Day 28, ng/ml)			
CON	2.0 ± 0.3 (47)	8.3 ± 0.3 (75)	8.1 ± 1.2 (15)
RPM	2.5 ± 0.5 (48)	8.0 ± 0.4 (81)	6.0 ± 0.9 (9)
P-value^3^	0.70	0.83	0.21
Overall^4^	2.3 ± 0.3 (95)^a^	8.1 ± 0.2 (156)^b^	7.3 ± 0.8 (24)^b*^

^1^Treatments: Control (CON) = feeding with 60 g of dried distillers grain (1.87% Met of MP); and Rumen-protected methionine (RPM) = 21.2 g of rumen-protected methionine (Smartamine M, 2.34% MET of MP) + 38.8 g of dried distillers grain.

^2^Data are presented as mean ± SEM.

^3^P–values for comparisons between CON and RPM.

^4^Mean ± SEM values with different superscripts in the same row are different (P < 0.001) or similar superscripts with an asterisk (*) tended to be different (P = 0.06).

The concentration of PSPB was not affected by RPM feeding (Table 5). There were similar concentrations of PSPB in CON and RPM treatments in cows diagnosed not pregnant on Day 28, pregnant cows at all examinations, and in cows that were pregnant on Day 28 but subsequently lost that pregnancy by the Day 61 examination (Table 5).

Cows that remained pregnant through the Day 61 after AI had greater serum P4 concentrations at Day 28 than cows that were not pregnant on Day 28 (determined by serum PSPB concentration; P < 0.001), and tended to have reduced (P = 0.06) P4 compared to cows that lost their pregnancy after Day 28. In addition, RPM feeding did not affect circulating P4 concentrations (Table 5) in cows that were not pregnant, pregnant, or pregnant on Day 28 but with subsequent pregnancy loss.

### Pregnancy diagnosis and pregnancy loss

To validly test our hypothesis, we only included synchronized cows in the analyses of reproductive traits (Table 6). Overall P/AI did not differ between treatments at Day 28, based on PSPB concentrations, or on Days 32, 47, or 61, based on ultrasonography. However, RPM treatment tended to reduce pregnancy loss between Days 28 and 61 (P = 0.08) and between Days 32 and 61 (P = 0.10).

**Table 6:** Overall effect of rumen-protected methionine (RPM) treatment on fertility responses and pregnancy loss in lactating dairy cows.

	Treatment^1^		
Item [% (n/total n)]	CON	RPM	P-value
Overall			
P/AI at 28 days	65.5 (91/139)	66.7 (96/144)	0.42
P/AI at 32 days	58.6 (82/140)	61.4 (89/145)	0.32
P/AI at 47 days	56.1 (78/139)	59.7 (86/144)	0.27
P/AI at 61 days	54.4 (75/138)	58.3 (81/139)	0.26
Pregnancy loss			
28 and 61 days	16.7 (15/90)	10.0 (9/90)	0.08
32 and 61 days	7.4 (6/81)	2.4 (2/83)	0.10
Primiparous			
P/AI at 28 days	63.5 (40/63)	66.7 (42/63)	0.35
P/AI at 32 days	58.7 (37/63)	60.3 (38/63)	0.43
P/AI at 47 days	56.5 (35/62)	57.1 (36/63)	0.47
P/AI at 61 day	54.8 (34/62)	56.5 (35/62)	0.43
Pregnancy loss			
28 and 61 days	12.8 (5/39)	14.6 (6/41)	0.41
32 and 61 days	5.6 (2/36)	5.4 (2/37)	0.50
Multiparous			
P/AI at 28 days	67.1 (51/76)	66.7 (54/81)	0.48
P/AI at 32 days	58.4 (45/77)	62.2 (51/82)	0.31
P/AI at 47 days	55.8 (43/77)	61.7 (50/81)	0.23
P/AI at 61 days	54.0 (41/76)	59.7 (46/77)	0.23
Pregnancy loss			
28 and 61 days	19.6 (10/51)	6.1 (3/49)	0.03
32 and 61 days	8.9 (4/45)	0.0 (0/46)	0.03

^1^Treatments: Control (CON) = feeding with 60 g of dried distillers grain (1.87% Met of MP); and Rumen-protected methionine (RPM) = 21.2 g of rumen-protected methionine (Smartamine M, 2.34% Met of MP) + 38.8 g of dried distillers grain.

When reproductive data were analyzed by parity, no effects of treatment were observed on P/AI in primiparous cows on Days 28, 32, 47, or 61. In addition, there were no effects of treatment on pregnancy loss in primiparous cows between Days 28 and 61 and Days 32 and 61 after AI.

Likewise, no effects of treatment were observed on P/AI in multiparous cows at Days 28, 32, 47, or 61. However, in multiparous cows, feeding RPM reduced pregnancy loss between Days 28 and 61 (19.6% vs 6.1%; P = 0.03) or between Days 32 and 61 after AI (8.9% vs 0.0%; P *=* 0.03).

### Embryo and amniotic vesicle size measurements on Day 33

Table 7 summarizes the measurements of amniotic vesicle volume and embryo size for all cows and for primiparous and multiparous cows analyzed separately. Overall, primiparous cows had greater amniotic vesicle volume (P = 0.01) compared with multiparous cows, but there were no parity differences for embryo volume. Cows fed with RPM tended to have greater embryo crown-rump length (P = 0.08), but had greater embryo abdominal diameter (P = 0.04), and embryo volume (P = 0.01), but no difference in amniotic vesicle volume compared to CON cows. When the measurements were analyzed by parity, the effects of RPM were more evident in multiparous than primiparous cows. There were no significant effects of RPM feeding in primiparous cows on amniotic vesicle volume, embryo crown-rump length, abdominal diameter, or overall embryo volume. In contrast, in multiparous cows, RPM treatment increased embryo size and amniotic vesicle. Cows fed with RPM had greater amniotic vesicle volume (P = 0.04), embryonic abdominal diameter (P = 0.02), and overall embryonic volume (P = 0.009), however, crown-rump length did not differ between RPM compared to CON cows.

**Table 7:** Effect of rumen-protected methionine (RPM) feeding on ultrasonographic morphometry of amniotic vesicle and embryo on gestation Day 33.

		Amniotic Vesicle	Embryo			
Treatment^1^^,^^2^	n	Volume (mm^3^)	n	Crown-rump Length (mm)	Abdominal Diameter (mm)	Volume (mm^3^)
Overall						
CON	63	542.6 ± 25.7	69	10.5 ± 0.2	5.5 ± 0.1	167.1 ± 6.0
RPM	80	594.9 ± 30.6	82	11.0 ± 0.2	5.8 ± 0.1	201.2 ± 10.6
P-value		0.27		0.08	0.04	0.01
Primiparous						
CON	30	617.1 ± 39.3	34	10.5 ± 0.2	5.6 ± 0.2	171.6 ± 7.6
RPM	36	596.0 ± 37.0	38	10.9 ± 0.2	5.7 ± 0.2	191.9 ± 14.3
P-value		0.67		0.21	0.61	0.38
Multiparous						
CON	33	479.4 ± 29.4	36	10.6 ± 0.2	5.3 ± 0.1	162.7 ± 9.2
RPM	44	593.9 ± 46.0	44	11.0 ± 0.2	5.9 ± 0.2	209.3 ±15.6
P-value		0.04		0.22	0.02	0.009

^1^Treatments: CONTROL = feeding with 60 g of dried distillers grain (1.87% Met of MP); and RPM = 21.2 g of rumen-protected methionine (RPM) (Smartamine M, 2.34% Met of MP) + 38.8 g of dried distillers grain.

^2^Data are presented as mean ± SEM.

## Discussion

Although previous studies have evaluated the *in vitro* effects of Met on bovine embryo development and function [19, 20], this is the first study that evaluated the effects of feeding RPM on milk production combined with reproductive efficiency of lactating dairy cows. In order to examine reproduction in sufficient numbers of cows, we utilized top-dressing of RPM in individual cows that were being fed a typical TMR on a commercial dairy. Similar methodology has been utilized in previous studies with daily top-dressing of rumen-protected choline [38] or daily feeding of conjugated linoleic acid in an automatic feeding system in the milking parlor [39]. This method allowed individual feeding of RPM and maintenance of cow as the experimental unit; although DMI in individual cows could not be measured or regulated using this approach. This approach increased statistical power, so effects of nutrition on reproductive efficiency of dairy cows could be evaluated; even though this first study on AA employing this approach utilized only 309 total cows, which would allow only 10% or more differences in P/AI to be statistically detected. The most important results from our study were: 1) An acute increase in plasma Met with a peak concentration at 12 hour after RPM top-dressing, 2) Increased milk protein percentage due to RPM, with no effects on milk protein yield, 3) Increased size of both amniotic vesicle and embryo on d 33 after AI in multiparous cows fed RPM, and 4) Similar P/AI between groups, but reduced pregnancy loss in multiparous cows fed RPM. The increased size of amniotic vesicle and embryo was consistent with the observed reduction in pregnancy loss from Days 28 to 61 or Days 32 to 61 in multiparous cows, although similar results were not observed in primiparous cows.

The effect of top-dressing RPM on the profile of plasma Met was relatively abrupt with a doubling in circulating Met from ~20 μM to ~50 μM at 12 hour after feeding and then returning to basal concentrations by 24 hour, using composites samples from 8 CON cows (n = 2 composites) and 12 RPM cows (n = 3 composites). A total of 125 different cows were analyzed for differences in plasma Met concentrations between CON (n = 56 analyzed in 19 different composite samples) and RPM (n = 69 analyzed in 25 different composite samples) cows. Use of composite samples allowed us to analyze more total cows in this study but was likely to reduce the variation in our results, since individual cows were not analyzed. The profiles of Lys and His, which are considered limiting AA in some dairy cattle studies, were not affected by RPM feeding, although there was a tendency for plasma His to vary during the day. Assuming that Smartamine M contains approximately 76% Met with bioavailability of 80% [40, 41], feeding with 21.2 g of Smartamine M would result in 12.9 g of bioavailable Met delivered per day. A second analysis of plasma AA was done evaluating cows only at 12 hour after feeding and this analysis confirmed the large increase in circulating Met that occurred after RPM feeding. Similarly, in mid-lactation cows, a study [42] determined the profile of plasma Met after supplementation with RPM using different products that differed in rate of degradation. Treatments were given as an oral bolus using either slowly or moderately degradable RPM. Cows fed the 30 g and 60 g slowly degradable RPM (11.9 and 23.8 g of bioavailable Met, respectively) peaked at 12 h after dosing, causing an increase of 70.3 and 133.9 μM. In non-lactating cows, oral dosing with 20 or 63 g of RPM (5.9 and 11.8 g of bioavailable Met) produced an increase to ~50 or ~60 μM, respectively, that peaked at 9–12 hour after RPM oral dosing [43]. Other experiments in lactating cows have also reported increased circulating Met after feeding with 10.6 g/d of bioavailable Met (16.5 to 27.2 μM based on a composite analysis of 3 pooled samples taken at 0, 2, and 6 hours after feeding done twice daily) [41] or 12 g/d of bioavailable Met (3.34 to 5.96 as a percentage of total AA at 2 hours after feeding done three times per day) [44]. Thus, top-dressing RPM once per day produces a dramatic but acute increase in circulating Met. This distinctive pattern may be important to consider when evaluating the effects of RPM feeding that were observed in this experiment.

Several AA that have been postulated to be limiting for milk protein production under specific dietary conditions in lactating cows including Met, Lys, and His. In this study, partially consistent with our first hypothesis, Met produced an increase in milk protein percentage, although effects on milk yield or milk protein yield were not significant in this study. This increase in milk protein percentage is in agreement with previous meta-analyses [16, 18], but lack of a response in milk protein yield is not consistent with responses that were previously reported. Consistent with most other studies there was no effect of RPM on overall milk yield [16, 44, 45]. A fairly large number of cows were evaluated in this study, however, one limitation of our study was that cows were sampled only once per month. Our results agree with other studies that observed a milk protein percentage response in cows fed with RPM once daily by top-dressing [46, 47] or with RPM incorporated into the TMR [48, 49]. A previous study by our research group [50] with RPM incorporated into the TMR found an increase of ~0.17% units and 40 g of protein yield. Other studies [41, 44] that performed top-dressing of RPM more than once daily seemed to observe greater production responses than observed in this study. It appears that delivery of RPM either mixed into the TMR or by periodic top-dressing can provide bioavailable Met to the mammary gland to increase milk protein production. However, future studies seem warranted to compare production and mammary gland responses to the acute and dramatic increase in circulating Met produced by once daily top-dressing compared to a more uniform delivery of RPM such as when it is incorporated into the TMR.

Our second hypothesis was that feeding RPM would have an effect on fertility of lactating dairy cows. Overall, fertility observed in this study for synchronized cows was excellent, with P/AI of 66% (187/283) at the 28 day and 60% (171/285) at the 32 day pregnancy diagnoses, utilizing only the cows that were synchronized by the Double Ovsynch protocol (92.2%; 285/309). Interestingly, we did not observe lower P/AI in multiparous cows as reported in some previous studies using the Double Ovsynch protocol [28, 29]. In the current study, P/AI was similar (P = 0.89) in primiparous [65% (82/126)] and multiparous cows [60% (105/157)]. Consistent with our study, some other studies using Double Ovsynch have also reported no effect of parity on P/AI [51–53]. Of particular importance to our second hypothesis, feeding RPM did not affect P/AI at any of the pregnancy diagnoses. Overall pregnancy loss in this study was 13% (24/180) from Days 28 to 61, which is similar to 12.8% reported in a summary of 14 studies (loss from Days 27–30 until Days 38–50) [54] and to 12% (loss from Days 27–40 until Days 56–90) recently reported in a summary with ~ 24,000 pregnancies at the first diagnosis from 46 recent studies [55]. Although, other studies have reported greater pregnancy loss in multiparous than primiparous cows [56], we found no differences between parities in this study. Nevertheless, pregnancy loss was greater in multiparous cows for CON compared to RPM cows from 28 to 61 days or from 32 to 61 days. Thus, no effects of RPM feeding were found on P/AI, although the small number of pregnancy losses (n = 24) was affected by treatment. In addition, one aspect to be mentioned is that generally cows carrying twins have greater pregnancy loss [57], but in this study none of the twin-bearing cows (CON = 3; RPM = 1) lost the pregnancy between Days 28 to 67 after AI, thus they had no influence in the final analysis.

Additionally, to evaluate embryo development we measured the concentrations of PSPB on Day 28 and the size of the amniotic vesicle and embryo on Day 33 after AI. As reported with other pregnancy-associated glycoproteins (PAGs), PSPB, also known as PAG1, is released by binucleate giant cells into the maternal bloodstream [58, 59] and detection of this protein has been used as the basis for pregnancy diagnosis [59–61]. The exact functions of PSPB are not clear, but it may be important for placental development and it has been used as a marker for placental function and fetal viability [62]. We hypothesized that feeding RPM would accelerate embryo development, increasing the size of the amniotic vesicle and embryo, with a corresponding increase in the circulating concentrations of PSPB. Overall, pregnant cows had higher PSPB concentrations compared to cows that subsequently lost their pregnancy. However, there were no treatment effects on PSPB concentrations on Day 28 after AI, even when divided into different pregnancy status groups. On the other hand, cows fed with RPM had greater size of embryo on Day 33 after AI. Similar to the effect of Met on pregnancy loss, we did not observe the effect of Met feeding on embryo/amniotic vesicle size in primiparous, but only in multiparous cows. Few studies have measured the size of embryo/fetus to evaluate embryo development in cattle [37, 63, 64]. In lactating dairy cows, a study evaluating the effect of slow-release bovine somatotropin (bST) on embryo development and fertility demonstrated that treatment with two doses of bST increased the size of the amniotic vesicle and embryo/fetus size at Day 34 and 48 after AI [65], but no effects were observed in PSPB concentrations on Day 28. Our results indicated that cows fed with RPM may have improved embryo development producing a larger size of embryo, but these cows did not have greater PSPB concentrations. Thus, embryonic size and amniotic vesicle size were increased by RPM in multiparous but not primiparous cows, consistent with the RPM-induced reduction in pregnancy loss in multiparous cows.

The mechanisms that underlie the increased size of amniotic vesicle and embryo at Day 33 after AI and reduced pregnancy loss by feeding RPM in multiparous cows remain unclear. As discussed below, the effects of Met could be at several stages of embryonic development and could be related to multiple biochemical pathways caused by inadequate Met such as an overall reduction in embryonic protein production, reduced activity in the one-carbon pathway potentially leading to reduced DNA methylation, or reductions in embryonic polyamines. One aspect to be considered is that multiparous cows may be more deficient in Met than primiparous cows, since greater milk protein production and milk yield could result in a greater requirement for Met. Analysis of control samples (Fig 3), indicated that multiparous cows had reduced circulating Met, compared to primiparous cows. Nevertheless, there was no observed effect of parity on circulating Met in cows fed Met. Thus, inadequate circulating Met concentrations in multiparous cows may be limiting intrauterine Met concentrations leading to delayed embryonic development and the observed reduction in embryonic size. Our research group has recently shown that multiparous cows have a larger uterine size and reduced fertility when compared to primiparous cows [35]. In addition, we have observed that Holstein heifers also have variation in uterine size and that recipient heifers with a larger uterine size are at a greater risk for pregnancy loss [66]. There is a dramatic increase in concentrations of AA in the uterine histotroph during early pregnancy in sheep [12] and cattle [10], including a large increase in Met, and this may be critical for optimal embryonic development. It is possible that cows with a larger uterine volume may have reduced AA concentrations in the uterine histotroph and potentially a greater AA requirement during the elongation and early placentation phases of pregnancy.

Alternatively, RPM feeding may lead to changes in the early embryo that are subsequently manifest in later stages of pregnancy. Recently our research group demonstrated that Met feeding did not affect fertilization and gross morphological quality of Day 7 embryos [67], but the diet rich in Met dramatically altered embryonic gene expression, in particular leading to reduced expression of many specific genes related to embryo development (e. g. VIM, IFI6, BCL2A1 and TBX15) and immune response (e. g. NKG7, TYROBP, SLAMF7, LCP1 and BLA-DQB [21]. An *in vitro* study [19] reported a surprisingly low Met requirement (7 to 21 μM) for development of morphologically normal bovine embryos. However, treatment of *in vitro*-produced bovine embryos with ethionine (a Met anti-metabolite) impaired embryo development at the blastocyst stage and addition of S-adenosylmethionine could partially restore embryonic development in the presence of ethionine [20]. Recently, an *in vivo* study reported that cows fed RPM produced early embryos with lower DNA methylation, but greater lipid content suggesting that Met feeding may affect energy metabolism and consequently, embryo survival [22]. Furthermore, in others species, Met metabolism can be involved in the synthesis of polyamines [68, 69]. Reduced polyamines is associated with impairments in early embryo development, attachment and growth of extraembryonic structures and placenta, and in steroidogenesis [70–72] highlighting other potential pathways that may cause pregnancy loss in animals with insufficient Met. Therefore, adequate Met may be required for correct embryo development, due to effects on DNA methylation and embryonic gene expression, general protein synthesis pathways, or specific metabolic pathways, such as polyamines, that may be crucial for optimal progress of the pregnancy.

In conclusion, this study demonstrated that top-dressing can be used as an experimental method for evaluating the effect of specific nutritional components on production and reproduction of lactating dairy cows on a commercial dairy farm. Daily top-dressing of RPM produced an acute increase in circulating Met and a small increase in percent milk protein. In addition, feeding with RPM was found to improve embryo development and reduce pregnancy loss, however, these reproductive effects were only observed in multiparous cows. Although the number of pregnancies is small and results should be interpreted with caution. Future research is warranted to confirm these reproductive effects as well as investigate the mechanism(s) responsible for these effects of feeding Met.
